# Who Benefits—Or Does not—From South Africa’s Old Age Pension? Evidence from Characteristics of Rural Pensioners and Non-Pensioners

**DOI:** 10.3390/ijerph13010085

**Published:** 2015-12-25

**Authors:** Margaret Ralston, Enid Schatz, Jane Menken, Francesc Xavier Gómez-Olivé, Stephen Tollman

**Affiliations:** 1 Department of Sociology, Mississippi State University, P.O. Box C, Mississippi State, MS 39762, USA; 2 Department of Health Sciences, University of Missouri, 535 Clark Hall, Columbia, MO 65211, USA; schatzej@health.missouri.edu; 3 Institute of Behavioral Science, University of Colorado Boulder, Boulder, CO 80309-0483, USA; menken@Colorado.edu; 4 MRC/Wits Rural Public Health and Health Transitions Research Unit, School of Public Health, Faculty of Health Sciences, University of the Witwatersrand, Johannesburg 2193, South Africa; F.Gomez-OliveCasas@wits.ac.za (F.X.G.-O); Stephen.Tollman@wits.ac.za (S.T.); 5 INDEPTH Network, P.O. Box KD213 Kanda, Accra, Ghana; 6 Umeå Centre for Global Health Research, Umeå University, Umeå 90001-90850, Sweden

**Keywords:** Africa, South Africa, pension, socio-economic status, self-reported disability, ageing

## Abstract

Social protection grants play a critical role in survival and livelihoods of elderly individuals in South Africa. Rarely is it possible to assess how well a social program reaches its target population. Using a 2010 survey and Agincourt Health Demographic Surveillance System census data we conduct multivariate logistic regression to predict pension receipt in rural South Africa. We find only 80% of age-eligible individuals report pension receipt. Pension non-recipients tend to be male, have poor socio-economic status, live in smaller households, be of Mozambican origin, and have poorer physical function; while older persons living in households receiving other grants are more likely to report pension receipt. We conclude that a reservoir of older persons exists who meet eligibility criteria but who are not yet receiving pensions. Ensuring that they and their households are properly linked to all available social services—whether for child or old-age social grants—is likely to have beneficial and synergistic effects.

## 1. Introduction

South Africans in rural areas are ageing in a complex context with high rates of income inequality, unemployment and an increasing burden of disease that includes endemic levels of HIV as well as a growing epidemic of non-communicable disease, particularly among those over age 50 [1,2,3,4]. In this context, social protection grants play a critical role in the survival and livelihoods of individuals and households [5,6,7,8]. South Africa has a strong commitment to addressing poverty and creating opportunities for economic security for all its citizens. The Constitution of the Republic of South Africa (Act No. 108 of 1996) states that all citizens have the right to appropriate social assistance from the government. Older persons are vulnerable population and recent research calls for pension programs to be strengthened to combat poverty among the elderly population [9]. This paper focuses on take-up of the old-age pension, one of seven social protection grants that comprise the South African social safety net, and one of the most generous. The old-age pension is the only grant targeted at the elderly population.

We draw on data from Agincourt, a sub-district in Mpumalanga province in the northeastern corner of South Africa, formerly part of the Gazankulu homeland [2]. Low rainfall and high population density make this an unfavorable area for subsistence farming; the population has low levels of education and high rates of unemployment and migration to urban areas in search of employment. Labor migration is particularly concentrated among men aged 35 to 50 years; nearly 60% of men in this age group live outside the study area for more than 6 months per year [10]. While not as concentrated above age 50, a significant proportion of older men continue to live outside of the area for work, although the percentage drops with increasing age. When this reality is coupled with the fact that women live longer than men, it results in a larger de-jure population of older women than men. As in much of rural South Africa, multigenerational households are common [2], with nearly 85% of persons over age 60 living in multigenerational households [11]. This complex context has resulted in a community largely reliant on migrant remittances and government social grants, particularly the old-age pension, to meet households’needs [8,12,13].

Given the importance of this means-tested pension to household livelihood, it is crucial to assess factors that are associated with being pension-eligible but not reporting receipt, and to examine barriers to receipt. Since we are not able to definitively differentiate between coverage—the number of persons formally covered by pension—*vs.* up-take—the proportion of eligible persons actually receiving benefits, we focus on age-eligibility to determine the denominator in our assessment of up-take. It is possible that not all persons aged 60 and above in Agincourt meet the other eligibility requirements; in fact, there may be a small proportion of those in the highest socio-economic status category who are ineligible. However, from our knowledge of the population in this site, we are confident that those who would be ineligible for economic reasons are limited [2,8,14].

We examine possible barriers to access by comparing individual and household characteristics of age-eligible pension recipients and non-recipients in the Agincourt population. We investigate whether there are differences in household economic status or social and human capital (e.g., nationality, education, resources available) associated with pension non-receipt that can be considered barriers to pension access. For a sub-sample of the population, we examine if health is related to pension-receipt among those age-eligible. We hypothesize that it is the poorest rather than the wealthiest age-eligible individuals who are not accessing the pension. While wealthier individuals may have private or other resources and thus not receive the state-funded pension, the poorest individuals and those with disabilities or limited physical functioning may need additional support to overcome application and access barriers to this resource. We address these issues through the following questions:

1. Are pension recipients better off than eligible non-recipients?
2. What household and individual characteristic are associated with pension receipt?

### 1.1. South Africa’s Old-Age Pension

While private and employment-based pension programs exist in South Africa for those who have had formal employment, for nearly three-quarters of South Africans, the means-tested non-contributory government sponsored old-age grant is the main source of income over age 60 [15]. This pension program was established in 1928 to benefit the white and colored populations. It was modestly expanded to the black population in 1944 [16]. Only in the early 1990s did the government begin extending pension access to the majority of black South Africans [14]. Historically, women became eligible at age 60, but until 2008 men became eligible at age 65. Between 2008 and 2010, male age-eligibility decreased incrementally to age 60. Pensions are restricted to individuals with South African identification documents (either citizen or permanent resident) who meet the means-test. In 2014, a single person qualified if income was <R61,800 per year (~$5340) and s/he had assets worth <R891,000 (~$77,000). Married people qualified if combined income and assets totaled less than double the single person amounts. Other household members’ income and assets are not considered. A person may work informally or formally and receive the pension as long as they meet the means-test.

Application for the pension must be made in person (or by a selected family member with proper documentation) at a South African Home Affairs office; these offices are generally situated in urban centers, which means travel, and potentially multiple trips, before pension receipt is possible. After approval, to receive funds, the pensioner must appear in person at a pension pay point in his/her village on a designated day each month. Although the pension pay points are located within villages, no applications can be made at the pay points [17]. The following documents need to be presented at the time of application: 13-digit bar-coded identity document (ID), proof of marital status (if applicable), proof of residence, proof of income, proof of assets including the value of property owned, proof of private pension (if any), a bank statement of the previous three months, if previously employed Unemployment Insurance Fund (UIF) or discharge certificate from previous employer, and if spouse died within the last five years, a copy of the will and the liquidation and distribution of accounts. The application, completed in the presence of a Social Security Administration officer, includes an interview to ensure the individual qualifies for the grant. It may take up to three months to process the application; if denied the applicant has 90 days to appeal to the national Department of Social Development [17].

### 1.2. Pensions and Their Impact

As of 2005, less than 1% of white older persons received a state-funded pension as compared to over 80% of black older persons [18]. Coverage in similar programs across Southern Africa range from 53%–87% [19]. Pension receipt significantly increases income in black South African households [20]. The monthly pension in 2010 was SAR1080 (approximately USD100), nearly twice the median per capita income for the black population [17]. The pension provides many households with access to credit markets, and many older women with a stable income for the first time in their lives [21]. While technically a cash transfer for older persons, extensive evidence points to the majority of elders, especially women, sharing the pension with family members [19,22].

There is evidence that the pension has protective effects for *all* other members of households [12]. Women are more likely to pool their pension income with household members and their pensions also has a greater effect on other household members’ health and wellbeing [5,23]. Still, pension receipt generally reduces stress for all adults within the household [24] and improves outcomes for children [12,23]. Similarly, Schatz *et al.* found that the pension played an important role in female-headed households affected by HIV by providing resources that buffered against the emotional and financial costs associated with HIV-related morbidity and mortality [13]. Yet, there is limited and mixed evidence that pension receipt directly and positively influences the health and wellbeing of older adults [25,26,27,28]. Case found evidence of pensions improving older persons health in rural South Africa [25]. Schatz *et al.* found that female pensioners, particularly in the first five years of pension receipt, reported better health than women who were not yet pension eligible [28]. Ardington and colleagues suggest that the pension mitigates the effects of crisis on older person’s wellbeing by reducing the financial and emotional impacts of an adult child’s death and the resulting carework for grandchildren left behind [24]. However, Lloyd-Sherlock and Agrawal found no association between pension receipt and hypertension, self-reported health or quality of life in a nationally representative South African sample [27]. While the evidence is mixed, there is an emerging consensus that pensions play multiple roles for individuals and their households—cash transfers to the poor and vulnerable, which can be used for individual health needs, and as a means of bolstering coping strategies of older people and their families.

## 2. Methods

### 2.1. Data

The Agincourt Health and Socio-Demographic Surveillance System (Agincourt HDSS), run by the MRC/University of the Witwatersrand Rural Public Health and Health Transitions Research Unit, has collected census data annually from all households in the Agincourt sub-district since 1992. In 2010, the site covered 27 villages—approximately 15,600 households and 89,000 individuals. In 2003, about one-third of households included at least one pension age-eligible individual, and 6% had two or more [29].

This study uses the Agincourt HDSS census and an abbreviated version of World Health Organization Study of global AGEing and adult health (WHO-SAGE) survey. The Agincourt HDSS census updates information on births, deaths, migration and household membership yearly. Other information regularly updated includes social grant receipt, educational attainment, and headship. Additional information is captured through occasional add-on census modules, e.g., socio-economic status of households. In 2010, the Agincourt HDSS collected health and wellbeing data on persons over the age of 50 through an abbreviated WHO-SAGE survey*.* The instrument contained two modules adapted from the full WHO-SAGE questionnaire: Health Status and Activities of Daily Living (following the WHO Disability Assessment Scale version II (WHODAS-II) model), and Subjective Wellbeing [30]. In 2010, 9431 individuals were 50 years or older and permanent residents of Agincourt, of these 4915 individuals were aged 60 or older and had complete census records. Approximately 60% of the target population completed the WHO-SAGE questionnaire with only 0.4% refusing. Others were either not found (35%), ineligible (4%) or dead (1.6%). The resulting WHO-SAGE sample contains 6025 individuals age 50 and above, about 25% male and 75% female, of these 3662 are pension age-eligible individuals, *i.e.*, those aged 60-plus.

### 2.2. Variables

#### 2.2.1. Pension-Receipt

The South African pension is means-tested, yet the majority of rural black South Africans have household incomes and assets well below the test line. While there is no current income data available for this sample, Case and Menendez conducted a study in the site in 2002 that found that the total household income averaged R1403 per month in non-pensioner households, and R1884 in pensioner households, with differences largely due to the pension income itself. Even with inflation and a near doubling of the pension amount from R570 in 2002 to R1080 in 2010, it is clear that the majority of older persons in the site would easily meet the means-test, which was the equivalent of salary of R5390 per month. 

Further evidence of the importance of pensions to older persons’ households is the fact that in 2010, 42.5% of older adults living in the study site did not have a currently working adult child as a household member, which means the pension income was likely the primary or only income in the household. In 2010, a question on pension receipt was included in the census for the first time. As shown in Table 1, fewer than 8% of individuals reported receiving the pension prior to age 60; the percentage then increased sharply, with over 80% of those 60 and over reporting receipt of either the old-age or disability grant. (individuals aged 60-plus may have been receiving the disability grant before becoming age-eligible for the pension. They cannot receive both grants, but since the grants are equivalent in value, we count reporting of either among those who are age-eligible as “pension-receipt”).

**Table 1:** Percent reporting pension receipt by sex and 5-year age group, Agincourt HDSS and WHO-SAGE 2010; % (*N*).

	WHO-SAGE		Agincourt HDSS	
	Men (*N* = 1530)	Women (*N* = 4473)	Men (*N* = 3547)	Women (*N* = 5826)
Age Groups				
50 to 54	3.3% (7/214)	2.2% (18/816)	1.2% (11/907)	1.5% (19/1283)
55 to 59	11.1% (27/244)	10.7% (80/750)	5.8% (46/792)	9.5% (102/1070)
60 to 64	73.9% (198/268)	79.3% (518/653)	53.8% (331/615)	75.2% (627/834)
65 to 69	84.3% (193/229)	84.4% (499/591)	72.9% (274/376)	81.5% (566/694)
70 to 74	88.1% (215/244)	86.8% (488/562)	81.2% (315/388)	84.0% (553/659)
75 plus	83.1% (275/331)	86.1% (951/1104)	80.0% (375/469)	84.6% (1088/1286)
% of 50–59 reporting pension	7.4%	6.3%	3.4%	5.1%
% of pension eligible (60+) reporting pension	82.2%	84.4%	70.1%	81.6%

#### 2.2.2. Possible Socio-Demographic Related Barriers to Pension Access 

Using census data, we consider the following socio-demographic variables: Education, marital status, employment status, nationality of origin, and a number of household level factors including household assets (a proxy for household socio-economic status), household structure, and the presence of other grants in the household. Education is categorized as no formal or some education. Marriage unions in this area may be traditional, civil, or polygamous (a small minority). *Marital status* is dichotomized into currently in partnership (civil or traditional marriage) or not (never married, separated, divorced, or widowed). *Employment status* is not asked in every year of the census. The module conducted closest to 2010 occurred in 2008. Employment status questions ask if the respondent is currently working, and then asks their primary occupation; the response options include both formal occupations and informal income generating activities. The variable is coded as *currently working* or *not*. The majority of those not working had retired and was not looking for work.

About one-third of the Agincourt population is of Mozambican origin. Most came to the Agincourt area as refugees during and after the Mozambican civil war, from the mid-1970s to late 1980s. *Nationality of origin* captures self-identification as South African or Mozambican. Previous Agincourt research showed that self-identified Mozambicans are less well-off than the host South African population in terms of education, household assets, and child mortality [31,32]. Prior to 2004, Mozambican permanent residents were not eligible for social grants. The South African Constitutional Court ruled in 2004 that Permanent Residents (the status of most Mozambicans living in Agincourt) were eligible for social grants. Even before this ruling, many Mozambicans accessed pensions through extra-legal means [33].

To measure socio-economic status (SES) of the household in which the older persons is living, we use a household asset score derived from 34 variables collected in the 2009 census (including type and size of dwelling, access to water and electricity, appliances and livestock owned, and transport available) as our measure of *socio-economic status* (SES) [34]. The SES score was derived through principal component factor analysis. We also consider a number of household structure variables to capture the multi-generational nature of Agincourt households [2]. These include *household size*, the *percent of individuals in the household under age 15*, and the *presence of an adult (aged 15–49) reporting currently working in the household.* We also examine the influence of the *presence of grants in the household*. We look specifically at child grants and “other” grants (e.g., foster care grants, care dependency grants, disability grants, war veteran’s grants).

#### 2.2.3. Possible Health-Related Barriers to Pension Access 

We use health indicators from the WHO-SAGE survey. *WHODAS II* (World Health Organization Disability Assessment Schedule II) is a 0–100 scale that measures day-to-day functioning in six activity domains and is based on multiple questions. WHO constructed WHODAS II from the Agincourt survey data. *Self-rated health* was examined through the standard question, *In general, how would you rate your health today? Very good, Good, Moderate, Bad, or Very bad.* We dichotomize self-rated health into bad (moderate, bad or very bad) or good (very good or good).

### 2.3. Statistical Analysis

We first present descriptive statistics on pensioners and non-pensioners to explore the nature and strength of the relationship between each variable and pension receipt. We then use multivariate logistic regression to predict pension receipt from a range of individual and household factors. In addition, we investigate interactions between key variables. The majority of descriptive statistics and regression models make use of the entire Agincourt HDSS census population aged 60 and over; analyses that include health measures are limited to the WHO-SAGE sample.

## 3. Results

In the Agincourt HDSS census population about 30% of age-eligible men and 18% of age-eligible women did not report receiving a pension (Table 1). As already mentioned, few individuals report pension receipt prior to age 60. As expected, pension receipt jumps substantially for the 60–64 age group and then increases steadily through age 74, with a slight drop-off in the oldest ages. Three-quarters of those aged 60 years and over, responded to the WHO-SAGE survey in 2010. The percentage reporting pension-receipt in the WHO-SAGE sample is higher than in the general population for both men and women, but particularly for men; thus, the WHO-SAGE sample may underestimates pension non-receipt. Since the Agincourt DHSS is a census with a designated reporter for each household, it is possible that when a person over 60 is not at home, particularly if that person is a migrant, the person reporting on pension receipt may be less reliable than when the person over 60 is home, which may account for at least some of the difference in reporting between the WHO-SAGE and the census data.

Table 2 displays descriptive statistics for adults aged 60-plus in the Agincourt HDSS census by reported pension receipt status. Table 3 presents these same descriptive statistics by response-status of WHO-SAGE sample. The statistically significant differences between pensioners and non-pensioners on key covariates were determined by *t*-tests. 

Pensioners and non-pensioners differ significantly on the following variables: Those reporting pension receipt are more likely to be women, South African, non-working, and in households receiving other social grant(s). Examining data from the WHO-SAGE sample, those reporting pension receipt also report a better health profile, with a lower mean disability score and lower proportion reporting bad self-rated health. In terms of household structure, 9.0% of pensioners live in households with only other individual 60 years and older, compared to 10.3% of non-recipients. While they only represent a small number of households, twice as many pensioners lived in skip generation households (1.2%) compared to non-pensioners (0.6%). For both pensioners and non-pensioners three-generation household structure was most prevalent (72%, 74% respectfully). Living alone was less likely among pensioners (6.3%) compared to non-pensioners (7.0%). Household size significantly differed by pension status with pensioners having slightly smaller households compared to non-pensioners (7.2 *vs.* 7.6). None of the other household level-variables (presence of working adult in household or percent of household under 15) differed significantly by pension status. Both the Agincourt HDSS population and the WHO-SAGE sample are skewed, with a higher proportion of women than men. In 2010, the South African national mid-year population estimates for individuals 60 year and older had a distribution of 58% women and 42% men. The Agincourt HDSS population over age 60 is slightly more skewed than at the national level, with 66% women and 34% men. The sex-distribution of the WHO-SAGE is 73% female and 27% male.

**Figure 1:**
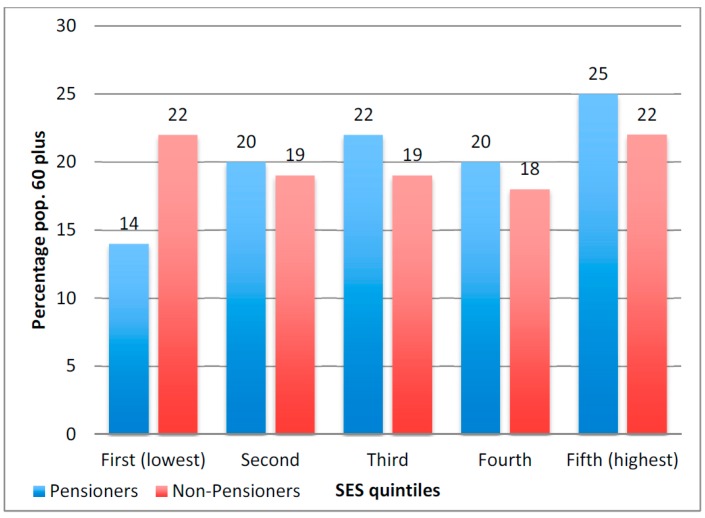
Distribution of socioeconomic status for persons 60 years and older by reported pension receipt, Agincourt HDSS 2010; *N* = 4915.

To include the most number of respondents, we utilize the whole Agincourt HDSS census population in Figure 1 and Table 4 Models 1–3; we use the WHO-SAGE sample Model 4 in Table 4 . Figure 1 shows SES distribution by pension receipt. The largest difference is the percent in the lowest SES quintile—fully 22% of non-pensioners *vs.* 14% of pensioners. The approximately 25% of the Agincourt population who report pension non-receipt (Table 1) are disproportionately the poorest. The Agincourt research team categorizes the three bottom quintiles as poor [31]. Using this measure, 60% of non-pensioners are poor, compared to 55% of pensioners.

**Table 2:** Description of adults 60+ by pension receipt, Agincourt HDSS and WHO-SAGE 2010.

	Pensioners (*N* = 3865)	Non-Pensioners (*N* = 1050)	*t*-test ^a^	Total (*N* = 4915)	Range
Female	68.4%	52.0%	−9.9 *	64.9%	0–1
South African	72.3%	53.2%	−12.0 *	68.3%	0–1
In partnership	42.2%	44.1%	1.1	42.6%	0–1
No formal education	69.3%	66.0%	−2.0 *	68.6%	0–1
Currently working	13.4%	36.8%	17.9 *	18.4%	0–1
Bad self-rated health ^b^	20.3%	23.6%	1.8	20.8%	0–1
Child grant in household	61.2%	56.4%	−2.9 *	60.2%	0–1
Other grant in household	7.9%	5.0%	−3.3 *	7.3%	0–1
Current working adult in household	62.6%	63.7%	0.7	62.8%	0–1
Only individuals 60 plus in household	9.0%	10.3%	1.3	9.3%	0–1
Skip generation household	1.2%	0.6%	−1.8 *	1.1%	0–1
Three generation household	72.0%	74.0%	−0.6	73%	0–1
Age: Mean (SD)	71.9 (8.4)	68.8 (8.8)	−10.6 *	71.3 (8.5)	60–106
Household size: Mean (SD)	7.2 (4.2)	7.6 (4.7)	2.8 *	7.3 (4.3)	1–39
Percent of household under 15: Mean (SD)	23.2 (17.5)	23.6 (17.1)	0.6	23.3 (17.4)	0–75
WHODAS disability Score II (0 = BEST); Mean (SD) ^b^	19.03 (19.3)	13.5 (19.6)	−8.3 *	17.8 (19.5)	0–100

Notes: ^a^ Compares differences in means of pensioners and non-pensioners (*t*-statistic reported); ^b^ Sample WHO-SAGE *N* = 3673 (non-pensioners = 554; pensioners = 3119); * *p* < 0.05 (two-tailed).

**Table 3:** Description of adults 60+ permanently in the Agincourt sub-district by response status to WHO-SAGE, 2010.

	Respondents (*N* = 3982)	Non-Respondents (*N* = 1339)	*t*-Test ^a^	Total (*N* = 5321)	Range
Pension receipt	84%	59%	−19.4 *	78%	0–1
Female	73%	42%	−21.5 *	65%	0–1
South African	68%	66%	−1.2	68%	0–1
In partnership	37%	53%	10.1 *	41%	0–1
No formal education	71%	64%	−4.4 *	69%	0–1
Currently working	12%	38%	22.1 *	18%	0–1
Child grant in household	59%	62%	2.5 *	60%	0–1
Other grant in household	8%	6%	−2.2 *	7%	0–1
Current working adult in household	60%	63%	1.0	60%	0–1
SES score: Mean (SD)	2.5(.42)	2.6(.43)	4.5 *	2.5	0.9–3.8
Age: Mean (SD)	72 (8.4)	69 (8.5)	−10.3 *	71 (8.5)	60–106
Household size: Mean (SD)	7 (4.2)	7.8 (4.4)	6.7 *	7.2 (4.3)	1–39
Percent of household under 15: Mean (SD)	23 (17.8)	24 (16.6)	1.4	23 (17.5)	0–75

Notes: ^a^ Compares differences in means of respondents and non-respondents; * *p* < 0.05.

Table 4 displays logistic regressions predicting pension receipt, clustered by household. Model 1 includes individual-level covariates. Model 2 adds household-level covariates and Model 3 includes an interaction between gender and nationality. Other interactions between key variables were tested but not found significant. Model 4 utilizes the WHO-SAGE sample and includes health measures.

**Table 4:** Logistic regression odds ratios and (95% CI) predicting reported pension receipt for adults 60+, Agincourt HDSS and WHO-SAGE 2010.

	Model 1	Model 2	Model 3	Model 4
Female	1.842 ***	1.799 ***	1.029	1.574 **
	(1.541–2.202)	(1.504–2.152)	(0.795–1.333)	(1.218–2.033)
South African	2.714 ***	2.568 ***	1.481 **	2.938 ***
	(2.309–3.190)	(2.157–3.056)	(1.155–1.899)	(2.366–3.648)
In partnership	1.676 ***	1.613 ***	1.598 ***	1.839 ***
	(1.389–2.023)	(1.332–1.952)	(1.321–1.934)	(1.432–2.361)
Age	1.031 ***	1.032 ***	1.033 ***	1.040 ***
	(1.020–1.042)	(1.022–1.044)	(1.022–1.044)	(1.025–1.054)
No formal education	1.129	1.164	1.150	1.005
	(0.957–1.332)	(0.985–1.376)	(0.971–1.362)	(0.803–1.257)
Currently working	0.334 ***	0.324 ***	0.318 ***	0.665 **
	(0.280–0.399)	(0.271–0.388)	(0.265–0.380)	(0.500–0.883)
SES score	-	1.427 **	1.446 **	1.543 **
		(1.153–1.766)	(1.171–1.786)	(1.174–2.028)
Household size	-	0.954 ***	0.952 ***	0.943 ***
		(0.935–0.972)	(0.934–0.971)	(0.918–0.968)
Child grant in household	-	1.760 ***	1.760 ***	2.025 ***
		(1.479–2.096)	(1.476–2.100)	(1.617–2.535)
Other grant in household	-	1.742 **	1.716 **	1.580 **
		(1.262–2.406)	(1.242–2.370)	(1.056–2.363)
South African * Female	-	-	2.527 ***	-
			(1.867–3.420)	
Bad self-rated health	-	-	-	0.823
				(0.650–1.041)
Disability score	-	-	-	0.990 ***
				(0.984–0.995)
N	4915	4915	4915	3685

Notes: Clustered by household; * *p* < 0.05; ** *p* < 0.01; *** *p* < 0.001 (two-tailed).

Relative odds remained largely the same across models with the exception, in Model 3, of those for female and South African. Pension receipt is positively associated with being in a partnership, higher age and SES score, and grant receipt. Being female and South African were strongly positive predictors of pension receipt in Models 1 and 2; their interaction is examined in Model 3. The main effect for gender is no longer significant; however, the main effect for nation of origin remains significant. The interaction term—showing that South African women differ from Mozambican women and all men—is highly significant and positive. Variables that have significant negative relationships to pension receipt, indicated by relative odds <1, include: Currently working, self-reported disability (WHODAS II) and household size. Odds of reporting pension-receipt are 33% lower for workers compared to non-workers (Model 4). Self-reported disability is a strong and consistent negative predictor of reporting pension receipt. The odds of reporting pension receipt are reduced by 1% for each unit increase in WHODAS II; however, reporting bad self-rated health was not a significant predictor of pension receipt.

**Figure 2:**
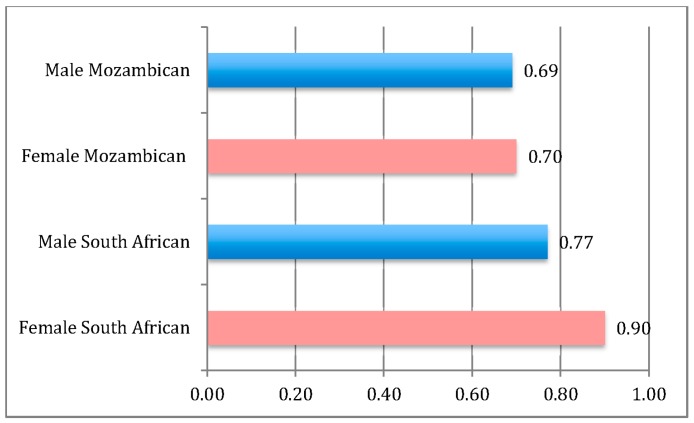
Predicted probability of receiving the pension by gender and nationality, Agincourt HDSS 2010; *N* = 4915. Figure derived from Model 3 in Table 3, with all variables but Female and South African set at their means.

Figure 2 shows the predicted probability of receiving a pension by gender and nationality when all other variables are held at their means. Female South Africans have the highest probability of reporting pension receipt while male South Africans and Mozambicans, male or female, do not differ significantly from one another.

## 4. Discussion 

This paper provides evidence for answering two central questions: (1) Are individuals who receive a pension better off than those who do not? (2) What household and individual characteristics are associated with reporting pension receipt?

For question 1, we assess up-take among age-eligible persons in Agincourt and find that: (a) about 25% report not receiving a pension; and (b) non-pensioners are disproportionately in the poorest sectors of the population. However, it is unclear whether pensioners are better off *because* they were able to access the pension, or if socio-economic disadvantage is itself a barrier to access. While our cross-sectional analysis cannot prove causality, it documents that it is unlikely that the majority of the 25% not receiving the pension are wealthy individuals foregoing pension due to lack of need since 60% of them are in the bottom three SES categories. Applying for the grant is likely to take multiple visits to a Home Affairs office [33]; in 2010 there were no Home Affairs offices in the Agincourt site. Twine and colleagues state that the cost of single trip by public transportation to the nearest public service offices was between R 5.00 and R 7.50 in 2002 (between USD 0.75 to USD 1). Due to inflation in transportation costs, the cost of the trip is now about USD 3.00. Applying for social grants requires knowledge, time, perseverance, and money, which may disadvantage the poorest in accessing grants [8]. We, therefore, believe that low socioeconomic status may be a barrier to applying for the pension. These results are even stronger in the WHO-SAGE sample, which is skewed toward women, partly because the men WHO-SAGE non-respondents are more likely to be working compared to the respondents (51% *vs.* 20%). The men who were available to be interviewed are also slightly poorer compared to the non-respondents (mean SES score of 2.6 *vs.* 2.7), and perhaps also have higher levels of disability.

In answering question 2, we find that gender, nation of origin, disability status, and other household level characteristics are also associated with pension receipt. There is evidence from research on child grant receipt that gender of household head may matter: Persons in female-headed households were significantly more likely to apply for child grants than those in male-headed households [8]. Women have been age-eligible at age 60 for the pension longer than men, which gives them time and knowledge advantage compared to men, and may help explain why South African women have higher odds of reporting pension-receipt compared to men. Because men’s age-eligibility was equalized to that of women, moving from 65 to 60 years between 2008 and 2010, there may be delays in their applying for the pension, which would account for their lower odds of receipt. However, it may also be that the poorest men, South African or Mozambican, have fewer social and economic resources than South African women to facilitate pension access. Further qualitative research is needed to understand differences in the processes through which women and men access the pension.

Mozambican men and women are less likely than South African women, even when controlling for SES and other important covariates, to report receiving the pension. A 2006 qualitative study of Mozambican women living in Agincourt showed that although all 30 respondents were legally eligible to receive the pension (the study took place after the 2004 Court judgment), barriers remained for accessing the grant [33]. Two-thirds *were* accessing the pension, but many had used extra-legal means prior to the court ruling (e.g., using a South African family member’s name to obtain identification documents). Similarly, Twine and colleagues found that Mozambicans used married names (to South Africans) or neighbors’ documents to secure child grants prior to the extension of rights to grants to permanent residents [8]. Among the one-third of older Mozambican women in the qualitative study not accessing the pension in 2006, the primary barrier was knowledge of the right to do so [33]. Furthermore many of the women thought the economic cost and physical energy required to obtain South African identity documents and subsequently the pension would better serve their families if used differently. Although not mentioned in this study, the need for an interview to “prove” one is eligible to receive the pension may contribute to reluctance to attempt to access the grant, particularly for Mozambicans whose citizenship status is less sure. Recent spates of xenophobia in South Africa may also influence the trust in government felt by those born outside South Africa. Taken together with our findings, this suggests that a special campaign involving the Department of Home Affairs, similar to one mounted to increase access to child grants in Agincourt and surrounding areas (described in more detail below) [8,35], may be needed to ensure that Mozambicans have the necessary documentation and know they are entitled to pensions.

Economic livelihood and health policies are often treated as separate issues; however they are intertwined [36]. In the more limited WHO-SAGE sample, self-reported disability is negatively associated with reporting pension receipt. The greater the disability a respondent reported, the less likely he/she was to report pension recipient. However, again it is unclear whether non-pensioners are worse off *because* they are unable to access the pension, or if their disability is itself a barrier to access. There is strong international support for the notion that pension receipt generally improves the health and mobility of older person [37,38]. But if disability, whether physical or social, is a barrier to travel to apply for a pension or to travel to receive the monthly funds, then the pension process as currently constituted may itself be a barrier for the disabled.

All households in our study include, by design, at least one pension age-eligible member. Of these, those in households with other social grants are more likely to report receiving a pension. We cannot tell which of the grants was applied for first; however, successful experience with one may lessen the burden of applying for an additional grant. With additional data points on grant receipt in Agincourt households, it will be possible to investigate the order of receipt of grants.

Research in Agincourt found that barriers to accessing child grants included lack of official documentation, education level, and distance to government service offices [8]. In response, Agincourt worked with the Department of Home Affairs to conduct a mobile registration campaign that targeted individuals eligible for child grants. There has not been a similar mobile campaign specifically aimed at registering those eligible for pensions. Although pension pay points are mobile and accessible in the community, they are not used as official application points [17]. If future research confirms distance to government service offices as a barrier to pension application/receipt, a mobile registration campaign may provide a way to increase take-up among eligible individual.

## 5. Conclusions

The old-age pension program is one of the most generous social-aid programs offered in South Africa (and indeed on the continent) and is a major instrument for redistributing resources to poorer households and communities in a highly unequal society. Many households depend on the grants; yet, those who have not been able to obtain them remain highly disadvantaged on several indicators. The pension is a means-tested benefit and therefore is more administratively cumbersome and more prone to bureaucratic subjective judgments, which might particularly disadvantage those born outside of South Africa or those who retain a lack of trust in government from the apartheid era. In this way the issue of “trust in government” may also play a non-negligible role in the explanation of non-receipt. Even if the means test is eliminated, as some have proposed [39], health and wealth barriers to accessing the pension may remain and need to be addressed in other ways. The issue of who has been unable to obtain a pension in South Africa, particularly in rural areas, is an important area of study. In the future, there may be additional data on older persons’ health, which would allow for a more in-depth examination of whether disability acts as a barrier to pension access, or if poorer health is a result of not accessing the pension.

The accomplishment of the South African government in providing a pension to three-quarter of elders in this rural setting is remarkable. However, this study calls attention to the other 25% and the need for additional data, as well as policies and interventions that focus on providing help to the poorest and most needy among them in accessing support for which they are eligible. While there are two data sources for this paper, the census data provide an important picture of all pension age-eligible persons’ households in the site, but are limited in that the respondent answering questions may not have been the older person him/herself. The WHO-SAGE data has the strength of providing more personalized data answered by the individual older person, but the data are skewed toward those more likely to be home-women, men who are not working or may have higher levels of disability. It will be important in future work to find and interview the types of individuals who are missing from the WHO-SAGE data to find out more from them about their pension and health status. These findings are based on a rare opportunity to assess the extent to which a major governmental intervention intended to improve public health and wellbeing is reaching its target population. They demonstrate the need for studies of this type in other settings and for other interventions. They also point to the need for strengthening engagement between researchers and policy-makers so that research findings may translate into improved policy and programs.
